# *Bacillus thuringiensis *Cry1Ca-resistant *Spodoptera exigua *lacks expression of one of four Aminopeptidase N genes

**DOI:** 10.1186/1471-2164-6-96

**Published:** 2005-06-24

**Authors:** Salvador Herrero, Tsanko Gechev, Petra L Bakker, William J Moar, Ruud A de Maagd

**Affiliations:** 1 Business Unit Bioscience, Plant Research International B.V., Wageningen University and Research Center, P.O. Box 16, 6700 AA Wageningen, The Netherlands; 2 Laboratory of Virology, Department of Plant Sciences, Wageningen University, Binnenhaven 11, 6709 PD Wageningen, The Netherlands; 3 Department of Entomology and Plant Pathology, Auburn University, Auburn, Alabama 36849, USA

## Abstract

**Background:**

Insecticidal toxins from *Bacillus thuringiensis *bind to receptors on midgut epithelial cells of susceptible insect larvae. Aminopeptidases N (APNs) from several insect species have been shown to be putative receptors for these toxins. Here we report the cloning and expression analysis of four APN cDNAs from *Spodoptera exigua*.

**Results:**

Suppression Subtractive Hybridization (SSH) was used to construct cDNA libraries of genes that are up-and down-regulated in the midgut of last instar larvae of beet armyworm, *S. exigua *exposed to *B. thuringiensis *Cry1Ca toxin. Among the clones from the SSH libraries, cDNA fragments coding for two different APNs were obtained (APN2 and APN4). A similar procedure was employed to compare mRNA differences between susceptible and Cry1Ca resistant *S. exigua*. Among the clones from this last comparison, cDNA fragments belonging to a third APN (APN1) were detected. Using sequences obtained from the three APN cDNA fragments and degenerate primers for a fourth APN (APN3), the full length sequences of four *S. exigua *APN cDNAs were obtained. Northern blot analysis of expression of the four APNs showed complete absence of APN1 expression in the resistant insects, while the other three APNs showed similar expression levels in the resistant and susceptible insects.

**Conclusion:**

We have cloned and characterized four different midgut APN cDNAs from *S. exigua*. Expression analysis revealed the lack of expression of one of these APNs in the larvae of a Cry1Ca-resistant colony. Combined with previous evidence that shows the importance of APN in the mode of action of *B. thuringiensis *toxins, these results suggest that the lack of APN1 expression plays a role in the resistance to Cry1Ca in this *S. exigua *colony.

## Background

During sporulation, *B. thuringiensis *(Bt) produces a crystal composed of one or more Cry proteins with toxicity against insects. The mode of action of the largest group of Cry proteins has been extensively studied and can be divided into four main steps: (i) Solubilization of the inclusion body to release the Cry proteins in their protoxin form, (ii) gut protease processing of these protoxins to an active toxin, (iii) binding of the active form to specific receptors in the midgut of the insect, (iv) membrane insertion, pore formation, and cellular lysis [1]. Any alteration in one of these steps could result in the development of resistance to one or several Cry proteins in a given insect population.

Development of insect resistance to insecticides is one of the most important problems of agriculture because it increases the costs of crop protection and reduces its productivity. More than 500 insect and mite species have been reported to develop resistance to one or more pesticides [2]. Although less in number, several cases of resistance have been also reported for *B. thuringiensis *insecticides in field populations as well as in laboratory selection experiments [3]. With the commercialization of transgenic crops expressing *B. thuringiensis *toxins (Bt-crops) the selection pressure has increased, with the consequent increased risk of resistance development. A high-dose/refuge strategy has been proposed and implemented in some cases to delay the development of insect resistance to Bt-crops. The effectiveness of this strategy mainly depends on the mode of inheritance of resistance and the initial frequency of Bt resistance alleles. Knowledge of the genes involved in Bt resistance will allow fast molecular screening for resistance gene frequencies in the field before or during use of a Bt crop. Furthermore, to determine if refuges or any other resistance management strategy are working, one should keep track of the frequency of resistance in field [4].

So far, the best-characterized mechanism of Bt resistance is the alteration of Cry protein binding to its receptors in the midgut of the target insect. This alteration has been reported in different populations of *Plutella xylostella, Plodia interpunctella, Heliothis virescens*, and *Spodoptera exigua *and also for several Cry proteins (Cry1Aa, Cry1Ab, Cry1Ac, and Cry1Ca) [3]. Aminopeptidase N (APN) and cadherin-like proteins have been characterized as candidates for Cry1 toxin receptors [1]. Gahan et. al, 2001 [5] studying the *H. virescens *(YHD2) resistant strain showed that the major gene for resistance to Cry1Ac in this strain was highly linked to the locus coding for a cadherin-like protein, and that this gene was disrupted in the resistant strain by the insertion of a retrotransposon. In 1994, Knight et al. [6] identified an APN as an insect receptor of the Cry1Ac protein. Since 1994 to today, more than 60 different APNs from different Lepidoptera have been sequenced and registered in databases showing the high diversity in isoforms. Other putative receptors for Cry toxins are proteins [7], glycoconjugates [8], and glycolipids [9].

Previous selection studies in the beet armyworm, *S. exigua *showed the ability of this insect to develop high levels of resistance (>75-fold at generation 35) to the Cry1Ca activated toxin [10]. Binding of this protein to brush border membrane vesicles (BBMV) from the midgut of the resistant insects was decreased 5-fold in affinity compared with the susceptible insects [10].

In our study, using Suppression Subtractive Hybridization (SSH), we compared *S. exigua *midgut gene expression between Cry1Ca-exposed and non-exposed susceptible insects and between resistant and susceptible non-exposed insects in order to identify genes that may be differentially expressed and therefore putatively involved in the insect response to toxin action or in resistance. Based on the SSH results, full cDNA sequences of four APNs were obtained. Northern blot analysis showed the lack of expression of one of the APNs in the insects from the resistant colony.

## Results

### Isolation of APN encoding cDNAs

In order to study genes that might be involved in the insect's response to intoxication by an active Cry toxin and/or in the mechanism of resistance to such a toxin, we took the approach of selection of cDNAs representing genes, which are differentially expressed in these different conditions. Suppression Subtractive Hybridization (SSH) was performed, in the first experiment with midgut cDNA from Cry1Ca-sensitive 5^th ^instar *S. exigua *larvae that had been raised on either control diet or on diet containing a sublethal dose of Cry1Ca protein during the entire larval life stage. Thus two pools of subtracted cDNAs were obtained and cloned, one presumably containing cDNAs representing genes with increased expression (library 1) and one representing genes with decreased expression (library 2) in response to toxin exposure, respectively.

In a second experiment, SSH was performed with midgut cDNA pools from Cry1Ca-sensitive and Cry1Ca-resistant 5^th ^instar larvae, both raised on diet without toxin. Thus two more pools of subtracted cDNAs were obtained and cloned, one representing genes with comparatively higher expression in sensitive larva (library 3) and one with lower expression in sensitive larva (library 4) as compared to resistant larva.

From each of the four libraries 96 clones were randomly picked and sequenced. Sequences were analyzed and overlapping or identical sequences were assembled into contigs. An in-depth analysis of the characterized sequences and the studies on the expression of the corresponding genes by microarray analysis will be published elsewhere. Homology searches with contig sequences resulted in the identification of a number of contigs encoding peptides with high homology to known lepidopteran Aminopeptidase N sequences and were selected for further studies.

In library 1 (higher expression in toxin-exposed larvae) we identified two single sequences of 375 and 436 base pairs, respectively. The first, designated contig 13 (APN2, following the classification of Nakanishi et al. [11]), encoded a peptide with high homology with *Lymantria dispar *APN2 [GenBank: AAD31184] amino acids 265 to 389 and almost equally high homology to a *Bombyx mori *APN [GenBank: BAA32140]. The second, contig 31 (APN4) showed highest homology to *Spodoptera litura *APN [GenBank: AAK69605] amino acids 173 to 296 and to a lesser extent to a second *B. mori *APN [GenBank: BAA33715]. The fact that the two encoded peptides were overlapping but not entirely identical in the overlap region, and were homologous to two different *B. mori *APNs, indicated that they might be fragments of two different APN cDNAs.

In library 3 (higher expression in sensitive larvae compared to resistant larvae) we identified 3 contigs with homology to APNs. Contig 82 (APN1) consisted of 6 identical copies of a 309 base pair fragment, while contigs 101 and 128 consisted of single fragments of 142 and 144 bp, respectively. All three sequences encoded peptides with high homology to different parts of *Helicoverpa armigera *APN1 [Genbank: AAK85538] (amino acids 19 to 123, 388 to 430, and 666 to 709 for contigs 82, 101, and 128, respectively) and to a lesser extent to a third *B. mori *APN [Genbank: AAC33301]. Although the three peptides were not overlapping, the high homology to single respresentatives of the APNs of *H. armigera *and *B. mori *indicated that they might be derived from one single *S. exigua *cDNA, different from the two cDNAs derived from library 1.

Overall results from SSH experiments provided the partial sequence of 3 different APNs (APN1, 2 and 4). Since previous work [11,12] suggested the presence of at least four different classes of APNs in Lepidoptera, a degenerate primer (Table 1) based on the known sequences of APN class 3 of other Lepidoptera was designed for the specific amplification of the *S. exigua *APN corresponding to class 3 in 3'RACE experiments. Sequences obtained from 3'RACE were employed for the design of a primer for the 5' end fragment amplification (Table 1).

**Table 1:** Sequence and localization of the primers employed for the isolation ofthe midgut aminopeptidases from *S. exigua *by RACE experiments

Gene name	Primer sequence (5' to 3')		Position in ORF
APN1	3'Race	TGCGCTTCGAAGATTGGCTCACGATA	2018–2044
	5'Race	CTTCGATGAATGCCGAGACGCTGGAC	2122–2097
APN2	3'Race	CTTTGCTGCTGGTGCTATGGAGAACTGG	906–932
	5'Race	ACAGGGCTGACTTCATTTCCGAACCATT	1067–1040
APN3	3'Race	ATGCGTGAYGAYATGTACGGTAT	496–518
	5'Race	GCCAGGAGATAAGTTGACATTTTTGGGG	779–752
APN4	3'Race	TGGCTTTCCATGTGAGCGATTTTGTGC	728–754
	5'Race	CACACCGATTTCTGCAGCATACGAGTGC	849–822

### Aminopeptidase sequence analysis

**Figure 1:**
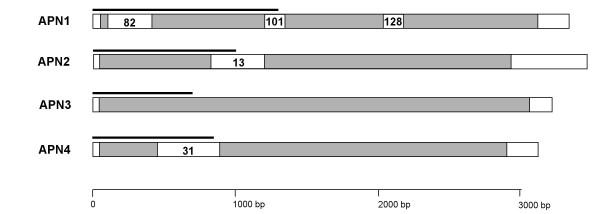
**Schematic representation of the four cloned APN cDNAs. **Gray bars correspond to the respective ORF. White blocks inside the gray bars represent the cDNA fragments coming from the SSH experiments and numbers correspond to the contig name. The black lines over each bar correspond to the fragment of RNA probe used in the Northern blots.

Using the primers described in Table 1, overlapping fragments from the 5'-and 3'-ends of the four APNs were amplified by RACE, cloned in pGEM-T Easy and sequenced. Open reading frames (ORF) of 3063, 2880, 3015, and 2853 bp were obtained for APN1 [GenBank: AY218842], APN2 [GenBank: AY218843], APN3 [GenBank: AY218844], and APN4 [GenBank: AY218845], respectively (Fig. 1). Protein products of 1021, 960, 1005, and 951 amino acids were predicted from the APN1, APN2, APN3, and APN4 cDNA sequences, respectively.

**Figure 2:**
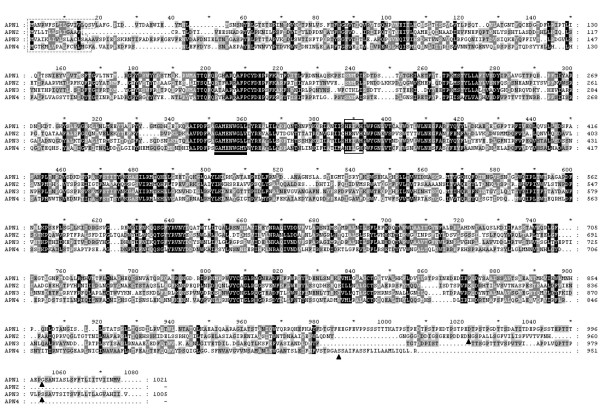
**ClustalX aligment of the deduced amino acid sequences from the four *Spodoptera exigua *aminopeptidases**. Dashed lines enclose the predicted signal peptides. Solid lines box the putative Zinc binding motives. The GAMEN motif is underlined. Predicted GPI-modification sites in the C-terminal sequence are indicated by the black triangles.

The predicted proteins were aligned using ClustalX as described in material and methods (Figure 2). Percentage of identity among the different APNs ranged from 36.7 between APN1 and APN3 to 23.3 between APN2 and APN3. Analysis of the N-terminal region of the predicted proteins using the SignalP program [13] indicated the presence of a signal peptide sequence in all four APNs (residues enclosed by dashed lines in Figure 2). All four APNs showed the presence of a zinc binding motif (residues enclosed by a solid line in Figure 2) and the GAMEN motif (underlined) characteristic of the gluzincin aminopeptidases and involved in their aminopeptidase activity [14]. Analysis of the amino acid sequences using the GPI Predictor Server [15] revealed the presence of a putative Glycosylphosphatidylinositol (GPI)-anchor site in the C-terminal end of all four aminopeptidases. The predicted GPI-modification sites were localized in the amino acid positions 1000 (APN1), 938 (APN2), 983 (APN3), and 931 (APN4).

**Figure 3:**
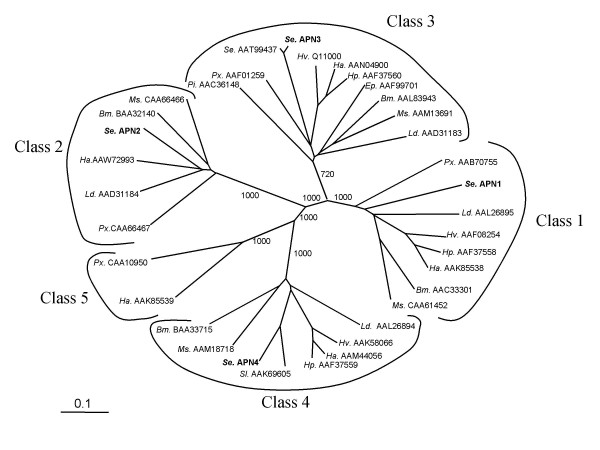
**Phylogenetic tree derived from a ClustalX alignment of all published lepidopteran midgut aminopeptidases**. Species name and GenBank accession number are shown for each protein. Numbers on the branches report the level of confidence as determined by bootstrap analysis (1000 bootstrap replicates). To clarify the figure, bootstrap values are shown only for the main branches. The scale bar indicates an evolutionary distance of 0.1 amino acid substitutions per position in the sequence. Species abbreviations: *Se: Spodoptera exigua; Ms: Manduca sexta; Ld: Lymantria dispar; Hv: Heliothis virescens; Ha: Helicoverpa armigera; Hp: Helicoverpa punctigera; Bm: Bombyx mori; Sl: Spodoptera litura; Px: Plutella xylostella; Pi: Plodia interpunctella; Ep: Epiphyas postvittana*

One allele each from all of the lepidopteran APNs deposited in GenBank so far (see methods section) were aligned together with these four *S. exigua *APN's using the ClustalX program. Phylogenetic tree construction showed that lepidopteran APNs group in five subfamilies (Figure 3). Alignment also shows that the four *S. exigua *APNs reported here each represent one of the larger four groups. Our APN3 is highly homologous to a *S. exigua *APN protein sequence deposited in GenBank during the course of our studies [GenBank: AAT99437]. Compared with the APN3 from the susceptible colony employed in this study, this APN showed 27 amino acid differences. Although degenerate primers were designed for the RACE of a *S. exigua *cDNA representative of the putative class 5, we were not able to obtain an amplification product corresponding to such an APN5. Bootstrapping analysis was performed in order to test the robustness of the generated tree. Bootstrap values of 1000 were obtained for all the main branches except for the one corresponding to the Class 3 APN. For this branch a bootstrap value of 720 was obtained, likely caused by the presence of the *P. interpunctella *APN [GenBank: AAC36148]. When this sequence was omitted from the alignment, bootstrap values of 1000 were obtained for all the five branches.

### APN expression analysis

**Figure 4:**
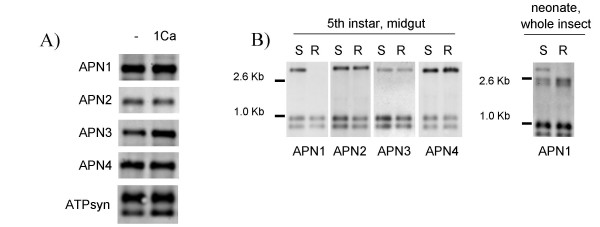
**Northern blot analysis of APN expression in 5^th ^instar midguts and whole neonate larvae of *Spodoptera exigua***. A. Comparison of expression of APNs in midguts from control (-) and toxin-exposed larvae (1Ca). An ATP synthase transcript (ATPsyn) was used as an internal control of RNA extraction and gel loading. B. Expression of APNs in midguts from fifth instar larvae (left panel) and whole neonate larvae (right panel) of the susceptible (S) and of the Cry1Ca-resistant (R) line. Higher molecular weight bands correspond to the different APN transcripts. Lower molecular weight bands correspond to the ATPsynthase transcripts.

The cDNA fragments of APN2 and APN4 originated each as a single fragment from a library putatively representing genes that were up-regulated in response to toxin exposure. However, when we compared expression of these genes as well as of APN1 and APN3 between guts of larvae exposed or not exposed to Cry1Ca toxin, by Northern blotting, we found no evidence for regulation of their expression by toxin exposure (Figure 4A).

The three cDNA contigs of APN1 were all derived from library 3, putatively representing genes that are expressed at a higher level in sensitive larvae compared to in resistant larvae. This suggested that APN1 was not, or at a lower level, expressed in the resistant line. In order to determine differences in expression levels of the APNs between the susceptible and resistant larvae, Northern blot analysis was performed with total RNA extracted from midguts of 5^th ^instar larvae of both colonies. Northern blot analysis showed that all four APNs are expressed in susceptible larvae (Figure 4B, "S" lanes). In agreement with the SSH results, no expression of APN1 was detected in batches of RNA from resistant larvae (around 10 larvae/batch), while the other three APNs were expressed at levels similar to that in susceptible larvae (Figure 4B, "R" lanes). Similar results were obtained with different batches of RNA (at least three) from the resistant insects and employing higher concentrations of RNA or in RT-PCR experiments specific for APN1 (data not shown), indicating that lack of APN1 expression is virtually complete in the resistant colony. Since most bioassays and selections for resistance are performed with the more toxin-sensitive neonate larvae in stead of the 5^th ^instar larvae used here, we also tested APN1 expression in (whole) neonate larvae from the sensitive and resistant colonies. Also here, APN1 expression was detectable in sensitive larvae, but not in resistant larvae (Fig. 4B, right panel).

## Discussion

Based on the results from SSH experiments and in one case by using degenerate primers (APN3), sequences of four different APN cDNAs from *S. exigua *have been determined. Sequence analysis of the predicted proteins showed the common structural characteristics that other lepidopteran APNs have, such as signal peptides, zinc binding sites, GAMEN motifs, and GPI-anchor sites. Oltean et al. [12] suggested the distribution of the different lepidopteran APNs in at least 4 homology groups following phylogenetic analysis of the sequences. Nakanishi et al. [11] called these groups class 1 to 4. Currently, with the addition of new sequences to GenBank, a new analysis clearly reveals the presence of at least five different classes of APNs in Lepidoptera. Definition of the fifth class is not only supported by the phylogenetic analysis of the sequences but also by the presence of a sequence from *Helicoverpa armigera *in all 5 branches [GenBank: AAK85538, AAW72993, AAN04900, AAM44056, and AAK85539]. Amplification of a *S. exigua *cDNA representing class 5 was attempted without success. Possible explanations could be that the degenerate primers used for the amplification of this APN were designed based on the consensus of only two sequences, and the selected primer sequences may not be conserved in a *S. exigua *APN5. Alternatively, *S. exigua *may not have a gene for the class 5 APN, or its expression in the midgut is too low to be picked up as cDNA.

SSH comparison between the susceptible and the resistant colonies suggested a lower expression of APN1 in the resistant insects compared with the susceptible insects. This difference in the expression was confirmed by Northern blot analysis (Fig. 4B), showing clearly the absence of expression of this APN in the resistant insects, both in 5^th ^instar larvae as well as in neonate larvae. APN has been described, together with cadherin-like proteins, as one of the proteins involved in the binding of Cry1A toxins to the midgut of several lepidopteran species [6,12,16-21]. Recently new evidence of the importance of APNs in the mode of action of Bt toxins was obtained by transformation of *Drosophila melanogaster *with the *M. sexta *class 1 APN [Genbank: CCA61452]. Expression of this APN in the midgut of the *D*. *melanogaster *larvae increased the susceptibility of these insects to Cry1Ac toxin [22].

In the case of Cry1Ca toxin, Luo et al. [23] using toxin affinity chromatography, detected and isolated a 106 kDa APN from *M. sexta *that bound to Cry1Ca. Although the entire sequence of this 106 kDa APN has not been published, its N-terminal sequence is nearly identical (9 out of 10 amino acids) to the sequence of the published *M. sexta *class 1 APN [GenBank: CAA61452] [24] and [GenBank: AAF07223] [25], and much more different from the *M. sexta *class 2, 3, and 4 APNs. This strongly suggests that at least one of the *M. sexta *Cry1Ca-binding proteins is a class 1 APN.

Agrawal et al. [26] cloned a *Spodoptera litura *class 4 APN cDNA, *slapn *[GenBank: AAK69605], and expressed it in insect cells, detecting binding of Cry1Ca toxin to the surface of the cells expressing this APN. More recent results showed that silencing of the expression of the *slapn *in *S. litura *by RNA interference (RNAi) through injection of a 756 bp dsRNA fragment, reduced the susceptibility of these insects to Cry1Ca [27].

So far, only one fully characterized gene has been associated with the development of resistance to Bt toxins in Lepidoptera. Gahan et al. [5] studied the *Heliothis virescens *YHD2 resistant strain and detected a strong linkage between the resistance to Cry1Ac toxin and the disruption of a cadherin gene. Unfortunately, since our *S. exigua *resistant line was lost during the development of this research, analysis of linkage between the resistance to Cry1Ca and the lack of APN1 expression could not be performed.

Data from SSH with resistant *vs *susceptible insects, support a possible role of the APNs in the mode of action of Bt toxins in Lepidoptera. Previous binding studies with the resistant line employed here, showed a 5-fold decrease in the binding affinity of Cry1Ca toxin to brush border membrane vesicles prepared with midguts from the resistant line. All these data together with previous work supporting the binding of Cry1Ca toxins to APN either in *M. sexta *or *S. litura*, strongly support the notion that lack of APN1 in *S. exigua *is responsible, at least in part, for resistance to Cry1Ca in this resistant line. Future work on the possible role of the APN in toxin binding, including heterologous expression or down-regulation by RNA interference strategies may further support this role.

## Conclusion

We have cloned and characterized four different midgut cDNAs from *S. exigua*, representing 4 out of 5 putative classes of lepidopteran APNs and have shown that one of these was not expressed in the larvae of a Cry1Ca-resistant colony. Combined with other evidence of the importance of APN in the mode of action of *B. thuringiensis *toxins these results suggest that the lack of APN1 expression plays a role in the resistance to Cry1Ca in this *S. exigua *colony.

## Methods

### Insects and tissue isolation

Cry1Ca-sensitive (parental strain of the Cry1Ca-resistant colony) and Cry1Ca resistant *S. exigua *larvae were obtained from laboratory colonies maintained at Auburn University [10]. The Cry1Ca-resistant population was selected with trypsinized Cry1Ca at 320 μg/gram diet most generations, including the generation preceding that from which eggs were obtained for gene expression studies [10]. Eggs were hatched and larvae reared on artificial diet at 28°C. Midguts were pulled from early 5^th ^instar larvae after cutting off the hindbody between the last two pairs of prolegs. Next, midguts were cut longitudinally and washed in phosphate-buffered physiological saline to remove gut contents. Midguts were stored at -80°C until further use.

### Toxin isolation

*Escherichia coli *expressing Cry1Ca was grown for protoxin production and protoxin was extracted, solubilized and trypsin-treated as described before [28]. Trypsinized (activated) toxin was dialyzed overnight against 50 mM sodium hydrogencarbonate pH9, 150 mM sodium chloride and quantified by SDS-PAGE. For feeding studies toxin solutions were diluted with PBS (Phosphate Buffered-Saline) to 250 ng Cry1Ca per gram diet and mixed with autoclaved artificial agar-based diet, which had been cooled to 55°C. Control diets merely contained equal amounts of PBS.

### mRNA isolation

Total RNA was extracted from whole 5^th ^instar midguts using TriPure Isolation Reagent (Roche Diagnostics, Almere) according to the protocols provided by the supplier. mRNA was purified from total RNA using GenoPrep oligo-dT beads (GenoVision, Oslo). Concentration of mRNA was calculated from OD_260 _and mRNA quality checked by agarose gel electrophoresis.

### Subtracted cDNA-fragment library construction

In order to isolate cDNA fragments representing mRNAs more abundant in midgut tissue of toxin-exposed or control diet-fed larvae, we used Suppression Subtractive Hybridization (SSH) [29] with the Clontech PCR-Select cDNA subtraction kit (Clontech, Palo Alto), according to the protocols provided by the supplier. Briefly, pools of mRNA were isolated as described above from 10 5^th ^instar beet armyworm midguts (exposed or unexposed to Cry1Ca toxin). cDNA was synthesized with AMV reverse transcriptase (first strand) and DNA polymerase I, and *E. coli *DNA ligase in the presence of RNase H followed by T4 DNA polymerase (second strand). cDNAs were digested with *Rsa*I, and appropriate linkers were ligated to the resulting cDNA fragments of the "tester" pool using T4 DNA ligase. cDNA fragment pools resulting from subtractive hybridization representing toxin-induced mRNAs ("exposed" pool as tester and "control" cDNA as driver) or for toxin-repressed mRNAs (reciprocal experiment) were further enriched with a "suppression PCR" step. Thus enriched cDNA pools were ligated into the vector pGEM-T Easy (Promega Benelux B.V., Leiden) and used for transformation of *E. coli *strain JM109.

In a second subtraction library construction experiment, cDNAs from 5^th ^instar larval guts of the Cry1Ca-sensitive line and of the Cry1Ca-selected line (both grown on diet without toxin), were used for subtractive hybridization. Pools containing cDNAs representing genes that were putatively expressed at a higher level (when cDNA of resistant insects was used as "tester") or at lower level (when cDNA of sensitive insects was used as "tester") in the resistant line compared to the sensitive line were separately ligated into pGEM-T Easy and used for transformation of *E. coli *strain JM109.

### Ampiflication of cDNA-5' and 3' fragments

For selected cDNA-fragments, both 3' as well as 5' cDNA fragments were amplified using the SMART RACE-kit from Clontech. For all cDNA-ends 5'-ready and 3'-ready cDNA pools were produced from reverse transcribed mRNA (using PowerScript reverse transcriptase) isolated from midguts of 5^th ^instar susceptible *S. exigua *exposed to toxin as described above. cDNA specific primers used for the 5' and 3' cDNA amplifications are listed in Table 1. Amplified cDNA-end fragments were purified using a QIAquick PCR purification kit (Qiagen, Benelux B.V., Venlo) and ligated into pGEM-T Easy (Promega Benelux B.V., Leiden).

### DNA sequencing and sequence analysis

DNA sequencing was performed by the dye-termination method using Bigdye terminator sequence mix (PE Applied Biosystems Benelux; Nieuwerkerk a/d IJssel) in an ABI 3700 automatic DNA sequencer. All oligonucleotides were obtained from Eurogentec (Brussels). DNA sequence homology searches were performed using the BlastX algorithm [30].

Comparison of the deduced amino acid sequences corresponding to the cloned APNs and phylogenetic reconstruction were performed using the ClustalX program [31]. A preliminary alignment was performed with the 61 different complete APNs protein sequences in the database. Based on the first alignment, redundant sequences, probably representing alleles from the same APN in the same insect species (identity higher than 95 %) were screened and a representative member for each class-species combination was selected for further analysis. A second phylogenetic reconstruction was performed with the 31 selected APNs and the four APNs isolated in current work. Phylogenetic reconstruction was obtained by the neighbor-joining method [32] together with bootstrap analysis using 1000 replicates. Kimura correction for multiple substitutions was applied [33].

### Northern blot expression analyses

Total RNA (1 μg) extracted from midgut tissue or whole neonate larvae was heat-denatured and applied to a 1% agarose gel in 2.2 M formaldehyde-MOPS buffer. After electrophoresis, the RNA was transferred overnight onto nylon membrane (Roche Diagnostics, Almere) and then UV crosslinked for 60 seconds. The labeled probe was prepared as follows. cDNA fragments cloned in pGEM-T Easy were amplified by PCR using M13 primers. The PCR products were purified and used as template for *in vitro *transcription using the SP6 polymerase in the presence of Digoxigenin-UTP, following the procedure described in the DIG RNA labeling Kit (Roche Diagnostics, Almere). Probe fragments of the APNs corresponded to the 5' end of the cDNA of each gene. As a control for equal loading of the Northern blots a probe corresponding to a cDNA fragment with homology to an ATP synthase obtained from the first subtraction library was used, for which microarray experiments showed no differential expression over a wide range of conditions (results not shown). The sizes of the probes were approximately 1300 bases (APN1), 1000 bases (APN2), 850 bases (APN4), and 700 bases (APN3, and ATP synthase) (Fig. 1). Hybridization and detection was performed following the instructions of the Dig Northern Starter Kit (Roche Diagnostics, Almere).

## Authors' contributions

SH participated in sequence analysis, expression studies, RACE PCR and phylogenetic analysis and manuscript preparation. TG participated in RACE PCR, sequence analysis and expression studies. PLB carried out the SSH experiments, sequence analysis and participated in RACE PCR. WJM performed insect selection, carried out the insect cultures and participated in the manuscript preparation. RAdM conceived of the study, participated in its design and coordination, and participated in manuscript preparation. All authors have read and approved the manuscript.
